# Editorial: Viral pathogenesis and host defense: understanding the missing links to combat disease

**DOI:** 10.3389/fcimb.2026.1806628

**Published:** 2026-02-27

**Authors:** Piyush Baindara, Akil Akhtar

**Affiliations:** 1 Animal Science Research Center, University of Missouri, Columbia, MO, United States; 2 National Swine Testing Center, University of Missouri, Columbia, MO, United States; 3 Emory Vaccine Center, Emory National Primate Research Center, Emory University, Atlanta, GA, United States

**Keywords:** antivirals, host-defense, host-directed therapies, immunity, infection, therapeutics, viral pathogenesis

**Figure 1:**
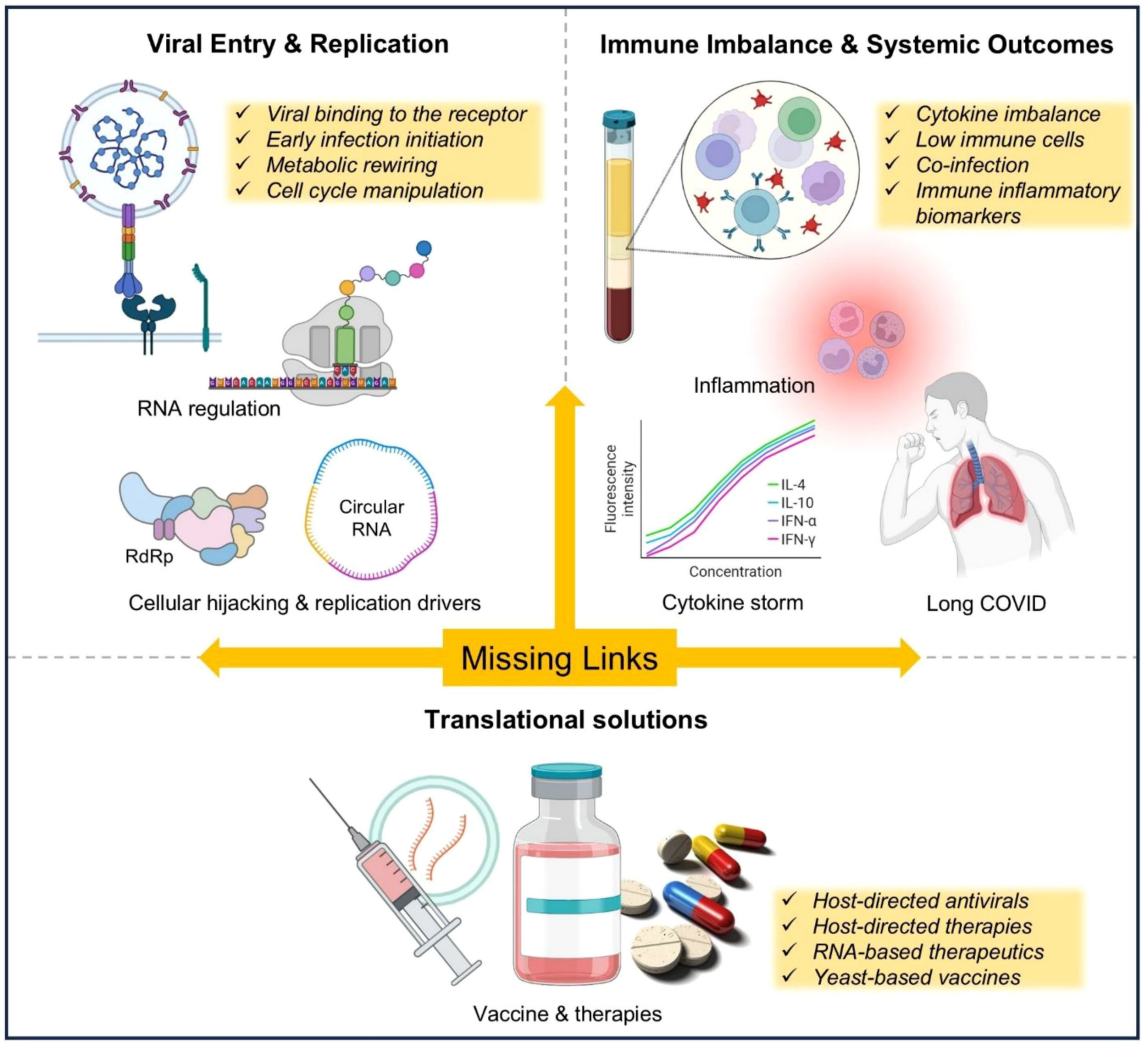
Conceptual framework linking viral pathogenesis, host defense, and translational opportunities. This schematic illustrates the progression from viral entry and cellular hijacking to metabolic and RNA-based regulatory mechanisms, culminating in immune imbalance, systemic inflammation, and long-term outcomes such as post-viral syndromes. The central “Missing Links” axis highlights mechanistic gaps connecting early infection events to clinical disease manifestations, while emphasizing translational opportunities including host-directed antivirals, RNA-based therapeutics, and next-generation vaccines.

Viral diseases remain among the most complex biological challenges of the present time, not simply because viruses replicate rapidly, but because they exploit core cellular pathways and reshape host biology. The true burden of infection lies in the “missing links” connecting viral entry to immune disruption, cellular hijacking to systemic pathology, and acute illness to long-term consequences (V’kovski et al., 2021). To combat disease effectively, the field must move beyond description toward a mechanistic understanding of host vulnerability and resilience, including the balance between protective antiviral immunity and harmful immunopathology (Blanco-Melo et al., 2020; Sette and Crotty, 2021). Overall, persistent immune dysfunction after viral clearance, alongside metabolic and RNA-based regulatory control of replication and immune evasion, highlights the need for next-generation antiviral strategies that strengthen host defense as well as target viral vulnerabilities (Taquet et al., 2022) (**Figure 1**). Altogether, published research and review articles in the present Research Topic collectively address critical mechanistic gaps linking viral entry, immune disruption, metabolic control, and long-term clinical outcomes.

One of the clearest lessons from recent viral outbreaks is that infection does not end when symptoms resolve. A central missing link in viral disease has been the trajectory of immune recovery. In this Research Topic, An et al. demonstrate that some moderate-to-severe COVID-19 survivors develop persistent lymphocytopenia, with reduced B cells, CD4^+^ T cells, and regulatory T cells even ~50 days after symptom onset. This highlights that immune recovery is not always complete and may contribute to ongoing susceptibility, inflammation, and long COVID outcomes. Next, innate immunity, often viewed as the immediate frontline of defense, is also deeply altered during severe viral illness. Next, Dagar et al. reveal that hospitalized COVID-19 patients exhibit impaired natural killer cell cytotoxicity, driven by disrupted granule trafficking and increased inhibitory receptor expression, with notable sex-associated differences that may contribute to unequal outcomes across populations. Together, these studies highlight that host defense is not only about mounting a response, but about restoring immune equilibrium after infection, a critical gap that future therapies must address.

Additionally, immune dysregulation shifts are unfolding within a broader landscape in which respiratory pathogen dynamics are changing in the post-pandemic era. An insightful study by Wang et al. demonstrates that respiratory pathogen patterns and immune responses differ substantially between children and adults, explained by cytokine profiling. These findings highlight age-specific differences in respiratory infection risk, supporting the need for tailored surveillance and therapies. The results also emphasize that host defense is shaped not only by individual immune response, but by population-level immune history, microbial ecology, and evolving viral circulation patterns.

Another important question in viral pathogenesis is why infection severity varies with environmental and physiological conditions. In this regard, Sun et al. address the long-standing observation that respiratory viral infections surge during colder seasons, demonstrating that low temperature enhances adenovirus replication by promoting intracellular alkalization and glycolytic flux. Overall, the study shows that host metabolism links environmental conditions to viral replication, making metabolic state a key driver of infection outcomes. It emphasizes that viral growth depends not only on the virus itself, but also on the cellular environment it infects.

Additionally, therapeutic innovation emerges naturally from mechanistic insights, particularly through host-directed strategies that disrupt viral dependence on essential cellular pathways. In this direction, Wang et al. explore flavonoid-rich compounds from *Forsythia suspensa* leaves as antiviral candidates against human adenovirus, showing that these compounds suppress infection by inducing G2/M cell cycle arrest and modulating key regulators such as CCNA2, AURKA, CHEK1, CCNB2, and CDC25A. This work highlights how natural products can influence cell cycle control to limit viral replication, offering host-directed antiviral pathways that may be less vulnerable to mutation-driven resistance than direct-acting antivirals.

At the earliest stage of infection, viral entry itself is a critical determinant of disease initiation. Qian et al. investigate goose astrovirus, an emerging avian pathogen, and identify HSPA5 as a candidate host membrane-associated binding factor using proteomics and structural docking. These findings provide a foundation for clarifying receptor-level interactions that govern infection initiation and may support future intervention strategies targeting viral attachment and entry.

Yet viruses do not succeed solely through entry. Their ability to evade host defense often depends on suppressing regulatory networks that coordinate antiviral immunity. Khan et al. provide striking mechanistic evidence that the infectious bronchitis virus QX hijacks host miRNA biogenesis machinery. The results demonstrate that the viral nucleocapsid protein interacts with ACE2, AGO2, and Dicer, disrupting pre-miRNA processing and weakening miRNA-driven antiviral defenses. This allows the virus to replicate more efficiently while promoting inflammatory cytokine responses and dampening anti-inflammatory control. Conclusively, the study highlights miRNA machinery as a key battleground in the host-virus interaction. Extending this RNA-centered view, Liu et al. highlight circular RNAs as emerging regulators of viral infection. CircRNAs can shape innate immune signaling, interact with miRNAs and proteins, and may serve as future biomarkers or therapeutic targets. Both host- and virus-derived circRNAs appear to influence immune evasion and viral replication, offering noncoding RNA regulation as an expanding frontier in host defense.

Importantly, viral pathogenesis extends beyond the respiratory tract and beyond classical infection endpoints. A recent study by Olivera et al. shows that HPV and *Chlamydia trachomatis* coinfection in men is linked to semen inflammation and reduced sperm quality, including lower motility and concentration. These findings highlight important fertility implications and support the need for reproductive screening in coinfected individuals. More broadly, the study reminds us that infection outcomes can extend beyond acute disease and involve long-term systemic effects. Moreover, Gong et al. provide a complementary clinical perspective, showing that immune imbalance can be reflected in simple blood-based inflammatory indices. In people living with HIV undergoing fracture surgery, an elevated Systemic Immune-Inflammation Index (SII) strongly predicted postoperative surgical site infections, outperforming traditional immune markers such as the CD4/CD8 ratio. These findings show how measurable immune-inflammatory states can serve as practical indicators linking immune suppression to real-world infectious outcomes.

Finally, a key priority in combating viral disease is preparedness: translating mechanistic understanding into vaccines and therapies before outbreaks escalate. Ganbold et al. review advances in vaccines and therapeutics for Severe Fever with Thrombocytopenia Syndrome (SFTS), emphasizing progress across platforms such as mRNA and viral vectors, alongside antiviral and immune-based treatment strategies. Their synthesis highlights both the promise of innovation and the urgency of bridging preclinical success to clinical deployment.

Taken all together, articles in this Research Topic illuminate viral pathogenesis as a multi-layered process: beginning with entry, shaped by metabolism, amplified through immune evasion, and extending into prolonged immune disruption and systemic consequences (**Figure 1**). They redefine host defense as more than an acute response, instead reflecting a long-term balance of immune restoration, regulatory control, and translational readiness. In this context, clarifying how viral mechanisms translate into host immune and clinical outcomes is essential for combating disease effectively. The future of antiviral therapeutics will depend on integrating mechanistic precision with clinical relevance, transforming fundamental insights into interventions that strengthen host resilience as much as they target viral vulnerability.
